# The Effect of Water-Soluble Polysaccharide from Jackfruit (*Artocarpus heterophyllus* Lam.) on Human Colon Carcinoma Cells Cultured In Vitro

**DOI:** 10.3390/plants9010103

**Published:** 2020-01-14

**Authors:** Adrian Wiater, Roman Paduch, Sylwia Trojnar, Adam Choma, Małgorzata Pleszczyńska, Paulina Adamczyk, Mateusz Pięt, Katarzyna Próchniak, Janusz Szczodrak, Jakub Strawa, Michał Tomczyk

**Affiliations:** 1 Department of Industrial and Environmental Microbiology, Maria Curie-Skłodowska University, ul. Akademicka 19, 20-033 Lublin, Poland; adrianw2@poczta.umcs.lublin.pl (A.W.); m.pleszczynska@poczta.umcs.lublin.pl (M.P.); paulinapolak2501@o2.pl (P.A.); kasiaproch@poczta.onet.pl (K.P.); szczo@poczta.umcs.lublin.pl (J.S.); 2 Department of Virology and Immunology, Maria Curie-Skłodowska University, ul. Akademicka 19, 20-033 Lublin, Poland; sylwia.trojnar@biophage.pl (S.T.); piet.mateusz@poczta.umcs.lublin.pl (M.P.); 3 Department of General Ophthalmology, Medical University, ul. Chmielna 1, 20-079 Lublin, Poland; 4 Department of Genetics and Microbiology, Maria Curie-Skłodowska University, ul. Akademicka 19, 20-033 Lublin, Poland; adam.choma@poczta.umcs.lublin.pl; 5 Department of Pharmacognosy, Faculty of Pharmacy, Medical University of Białystok, ul. Mickiewicza 2a, 15-230 Białystok, Poland; jakub.strawa@umb.edu.pl (J.S.); michal.tomczyk@umb.edu.pl (M.T.)

**Keywords:** *Artocarpus heterophyllus*, water-soluble polysaccharide, cytotoxicity, cytokines, nitric oxide, human colon tumor cells

## Abstract

Various phytochemical studies have revealed that jackfruit (*Artocarpus heterophyllus* Lam.) is rich in bioactive compounds, including carotenoids, flavonoids, volatile acids, tannins, and lectins. The aim of the study was to analyze the biological activity of water-soluble polysaccharide (WSP) isolated from jackfruit and to assess its immunomodulatory, cytotoxic, and anti-oxidative effects on human colon carcinoma cells in vitro. The neutral red (NR) uptake assay revealed no toxic influence of the polymer on the viability of tumor cells (HT29 and SW620). After 24 h and 48 h of incubation, the cellular viability was not lower than 94%. The metabolic activity of the cells (MTT) at the compound concentration of 250 µg/mL was higher than 92% in comparison to the control. WSP (250 µg/mL) exerted no significant effect on the morphology of the cells was determined by May-Grünwald-Giemsa staining. WSP changed nitric oxide (NOx) production by the tumor cells depending on the time of incubation and prior 2-h stimulation of the cells with *E. coli* 0111:B4 LPS. It significantly stimulated IL-1β production by the tumor cells. The IL-6 level increased but that of IL-10 decreased by a WSP concentration-dependent manner. No such effect was detected in SW620. The WSP had antioxidant properties. In conclusion, water-soluble polysaccharide isolated from *A. heterophyllus* exhibits significant biological activity towards many types of both normal and cancerous cells. Therefore, it may be considered as a useful agent in the protection of human health or in functional and dietary nutrition.

## 1. Introduction

*Artocarpus heterophyllus* Lam. (syn. *A. integrifolia* L., common name: jackfruit or jack tree), belonging to the family Moraceae, is a large evergreen tree native to the Western Ghats, India, and commonly cultivated in the South East Asia region [1]. The jack tree produces nutritious giant fruits, which are a popular food. One hundred grams of a ripe jackfruit contains the following nutrients: carbohydrates (18.9 g including starch, glucose, fructose, and sucrose), protein (1.9 g), fat (0.1 g), dietary fiber (1.1 g), total mineral matter (0.8 g), including calcium (20 mg), phosphorus (30 mg), and iron (500 mg), as well as vitamins (A, C, B and folic acid) and 77% of moisture [2]. The plant is widely used in traditional medicine as a remedy against skin diseases, including asthma, diarrhea, and fever (roots); worms, syphilis, ulcers, and wounds (leaves); anemia, asthma, cough, dermatitis, and diarrhea (leaves and stem bark) [1,3]. Phytochemical studies have revealed that jack tree is rich in bioactive compounds, mainly carotenoids, flavonoids, volatile acids, tannins, and lectins, which are responsible for several biological activities [4,5]. Crude extracts of various parts of the plant exhibited broad-spectrum antibacterial activity [3,6,7], including activity against cariogenic bacteria [8] and multidrug resistant *Staphylococcus aureus* [9]. A jackfruit seed-derived lectin, Jacalin, inhibits DNA viruses such as herpes simplex virus type II, varicella-zoster virus, and cytomegalovirus, and crude extracts from leaves have anti-hepatitis C virus activity [10,11]. Several investigators have reported the antioxidant activity of extracts from defatted jackfruit seeds, pulp, and dried mature fruits in in vitro assays [5]. Ko et al. [12] found that prenylflavonoids isolated from the plant are responsible for this effect and antiplatelet activity. It was also reported that aqueous extracts of *A. heterophyllus* leaves exhibited oral hypoglycemic activity [13] and one fraction of their ethanolic extract enhanced significantly the rate of wound healing in rats [14].

Plants are an inexhaustible source of natural polysaccharides [15]. To date, they have mainly been used in industry, but their biological activity is increasingly being investigated in terms of their application in medicine and pharmaceutical industry [15,16,17]. Despite their limited cytotoxicity, some of them show immunomodulatory activity significantly inhibiting the growth of cancer cells [18]. Moreover, activation and enhancement of the immune system by polysaccharides have been demonstrated not only in experimental systems in vitro but also in laboratory animals in vivo [18]. One of the important parameters associated with the immune system is the development of inflammation. It manifests itself as an increase in the level of pro-inflammatory mediators (cytokines, chemokines, etc.), adhesion molecules and, consequently, migration of leucocytes from the blood vessels into tissues and potential damage thereof [19]. IL-1β, TNF-α, and IL-6 are the most important pro-inflammatory cytokines. In turn, IL-10 acts as an anti-inflammatory factor. However, not only immune system-derived factors are responsible for inflammation but also molecules that are radicals. One of them is nitric oxide (NOx), which is released by nitric oxide synthase (NOS) during an inflammatory process. This molecule is important in proper functioning of biological systems, e.g., the nervous or immune systems. It is a highly reactive and unstable compound reacting with biological molecules or the oxygen radical contributing to its signaling or cellular effects [19,20,21]. It has been shown that polysaccharides from *Artocarpus heterophyllus* Lam. pulp exhibits antioxidant activities including DPPH• radical or •OH radical scavenging [22]. Generally, plant derived polysaccharides have many biological activities, including immunomodulatory, anti-tumor, or anti-oxidative effects [23,24]. Therefore, they can serve as adjuvant factors in basic, commonly used treatment regimens or as pro-health diet supplements.

Plant polysaccharides containing galactose most often belong to a cell wall storage polymer [25]. Galactose is a common component of pectic polysaccharides (rhamnogalacturonans). As (1→4)-*β*-linked linear galactan oligomers, they are usually attached as side chains to (1→4)-*α*-d-galacturonan chains, in which (1→2)-*α*-l-rhamnose residues are inserted. In rhamnogalacturonans, rhamnoses from main polymer chains are substituted by some oligosaccharides at position *O-4* [26]. Another type of the polymer is present in the fruits of dicotyledonous plants. This is type I arabinogalactan, i.e., (1→4)-*β*-galactan decorated with arabinose oligomers. Type II arabinogalactans are polysaccharides with greater complexity. Their core contains (1→3,6)-*β*-galactan branched with short galactosyl side chains, usually terminated with arabinofuranose, rhamnopyranose, and/or galactopyranose residues. The arabinogalactan chains are generally linked to a protein backbone [25,27]. A structurally diverse group of galactans occurs in red marine algae. The linear backbone of these sulfated polysaccharides consists of alternating units of 3-linked *β*-d galactopyranose and 4-linked *α*-d/l-galactopyranose and can be ramified by other neutral sugars such as glucose, arabinose, and xylose. These polysaccharides are represented by gelling galactans, agars, and carrageenans produced industrially from some *Rhodophyta* [25,28].

The aim of the present study was to analyze the biological activity of water-soluble polysaccharide isolated from *A. heterophyllus* fruits in terms of its immunomodulatory, cytotoxic, and anti-oxidative effects on the viability and proliferation of human colon carcinoma cells in vitro.

## 2. Results

### 2.1. Isolation and Structural Analysis of the Water-Soluble Polysaccharide

The purified water-soluble polysaccharide was extracted from *A. heterophyllus* fruits with a yield of 11.8% of the dried starting material. The specific optical rotation of the polysaccharide was +31° (*c* 1.0, H_2_O), whereas the values of viscosity were approximately 1.79 mPa·s. Monosaccharide analysis showed that this preparation consisted mainly of d-galactose (43.2%) and d-galactouronic acid (48.6%). In addition, d-xylose (4.4%) and d-glucose (3.8%) were found. The gas chromatography-mass spectrometry (GC-MS) analysis of permethylated alditol acetates showed that (1→4)-linked Gal*p* and GalA are the major chain constituents. The water-soluble polysaccharide dissolved in Milli-Q water exhibited a single and symmetrical peak on a PolySep™-SEC GFC-P 5000 column, indicating its homogeneity. The molecular weight (Mw) of the water-soluble polysaccharide was about 23 kDa.

**Figure 1:**
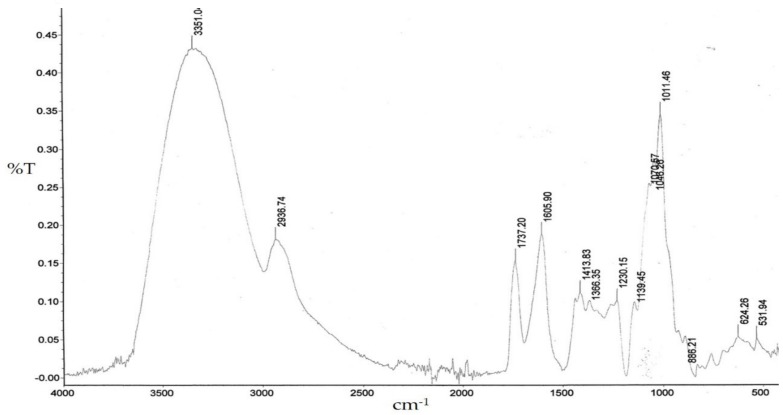
Infrared (FT-IR) spectrum of WSP from *A. heterophyllus* fruits.

The FT-IR spectrum of the lyophilized polysaccharide fraction from *A. heterophyllus* fruits is shown in Figure 1. An intense and broad absorption peak at 3351 cm^−1^ for O–H stretching vibrations, a peak at 2936 cm^−1^ for C–H stretching vibrations, and a broad absorption band in the region near of 1011 cm^−1^ for coupled C–O-C and C–C stretching and C–OH bending vibrations are characteristic of pyran ring structures [29,30].

Moreover, the absorption peak at 1737 cm^−1^ was attributed to the stretching vibrations of C=O of methoxylated carboxylic groups of GalA and the C=O stretching vibrations of carboxylic acid ions result in the peak at about 1606 cm^−1^. The last-mentioned absorption peak as well as the peak at 1414 cm^−1^ prove the acidic character of isolated polysaccharide. The high intensity of this peak indicates the predominance free COO^−^ groups. The calculated degree of esterification (DE) coefficient is around 40%. The absorption peaks at 886 cm^−1^ confirmed the existence of *β*-glycosidic bonds (most probably from Gal residues) [31]. Summarizing, the chemical analysis, the water-soluble polysaccharide extracted from *A. heterophyllus* fruits was a (1→4)-galacturonan with a molecular mass of approximately 23 kDa.

### 2.2. Water-Soluble Polysaccharide Activity in Cell Cultures in Vitro

The tested polymer concentration was from 0 to 250 µg/mL. In this range, it was possible to capture the biological activity of the WSP. In addition, the concentrations used are within the physiological range that can be used *in vivo*. 

**Figure 2:**
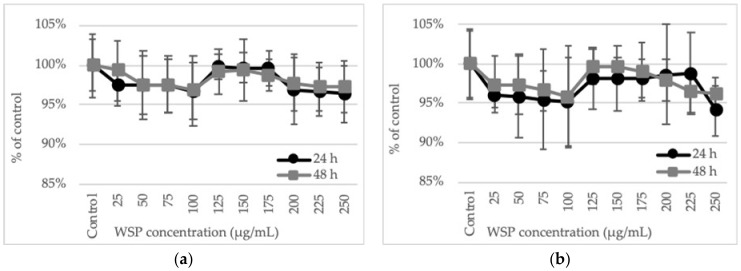
Effect of 24-h and 48-h treatment of HT29 (**a**) and SW620 (**b**) with WSP isolated from *A. heterophyllus*. Neutral red assay (NR). The results are presented as a percentage of the control arbitrarily set to 100%. The figure shows an average of three independent experiments.

The cells of both HT29 and SW620 human colon tumor lines did not show signs of viability loss in the NR uptake test after culture with *A. heterophyllus* WSP at concentration values up to 250 µg/mL. The viability did not drop below 94%. Similar results were observed after 24 and 48 h of incubation (Figure 2).

**Figure 3:**
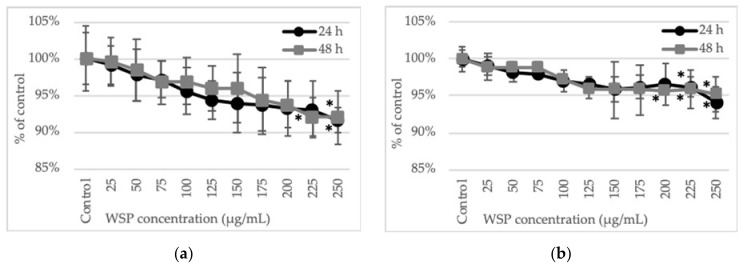
Effect of 24-h and 48-h treatment of HT29 (**a**) and SW620 (**b**) with WSP isolated from *A. heterophyllus*. MTT assay. The results are presented as a percentage of the control arbitrarily set to 100%. The figure shows an average of three independent experiments. * *p* ≤ 0.05 - cells treated with the plant polymer compared to the non-treated culture control.

When changes in cellular metabolism were studied by determination of the succinate dehydrogenase activity (MTT test), a gradual decrease in enzyme activity and thus cell viability was observed along with the increase in the concentration of the polysaccharide used. However, the metabolic activity of the cells at the highest WSP concentration (250 µg/mL) decreased no more than 8% in comparison to the control. Nevertheless, at the two highest WSP concentrations (225 and 250 µg/mL), a significant decrease in the cell viability was observed, in comparison to the control. The reactivity of the HT29 and SW620 cells to the *A. heterophyllus* WSP was similar. The sensitivity of the cells to the tested polysaccharide did not show significant differences after 24 and 48 h of incubation either (Figure 3).

**Figure 4:**
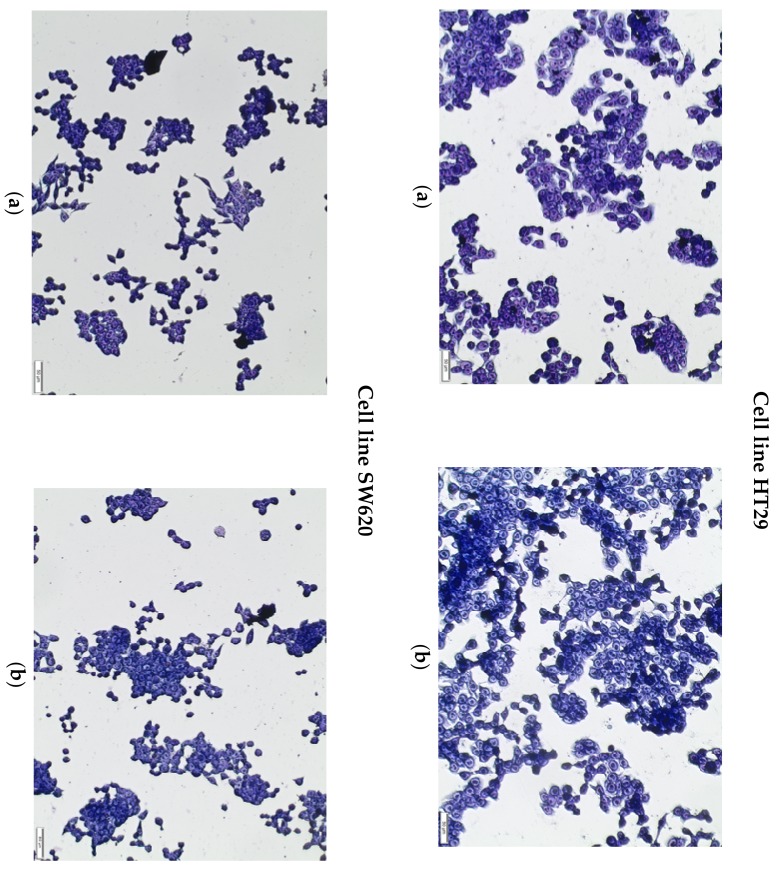
May-Grünwald-Giemsa staining of HT29 and SW620 cells after incubation with WSP isolated from *A. heterophyllus* at a concentration of 250 µg/mL. Image (**a**) represents control cells, while (**b**) represents cells treated with the polysaccharide for 24 h. Scale bar = 50 µm.

The results of the spectrophotometric tests were confirmed by the May-Grünwald-Giemsa staining (MGG) method, which indicated no morphological changes in the cells of both lines after incubation with the WSP at a concentration of 250 µg/mL (Figure 4).

**Figure 5:**
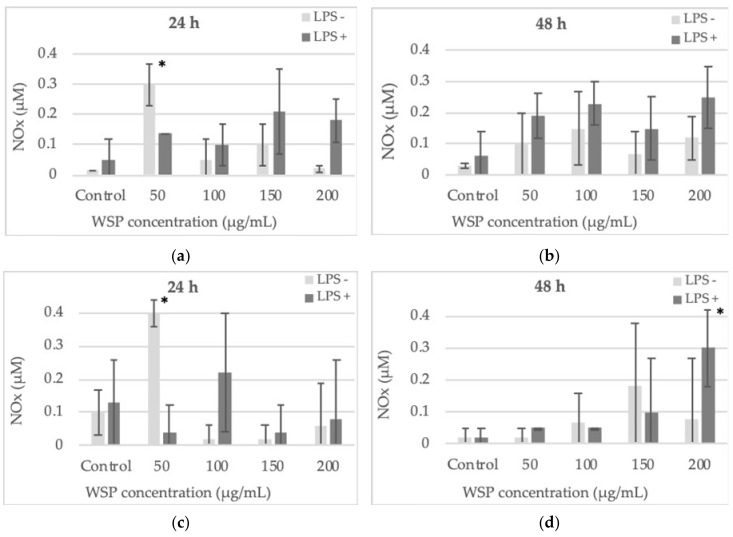
Nitric oxide (NOx) secretion in the culture of HT29 (**a**,**b**) and SW620 (**c**,**d**) cells during 24-h and 48-h incubation with water-soluble polysaccharide isolated from *A. heterophyllus*. Four concentrations of the polymer were used: 50, 100, 150, and 200 μg/mL. The analysis was performed using the Griess method. The columns and bars show the mean ± standard deviation (n=3), * *p* ≤ 0.05 - cells treated with the plant polymer compared to the non-treated culture control.

After 24-h incubation of the cells with the tested WSP, a significant increase in the nitric oxide (NOx) level was demonstrated in both cultures only at a low polysaccharide concentration (50 µg/mL) in conditions without pre-incubation of the cells with *E. coli* 0111:B4 LPS. In our tests, no possible contamination of the polymer with LPS was determined. After 48-h exposure of the cells to the WSP, the amount of NOx was higher than that obtained after the first day (first 24 h) of incubation. At the higher polysaccharide concentrations and earlier pre-incubation of the cells with *E. coli* 0111:B4 LPS, additional stimulation of NOx production was found, compared to the culture without the endotoxin pre-treatment. Both cell lines reacted similarly after 24-h and 48-h induction with WSP and after additional pre-incubation with *E. coli* 0111:B4 LPS (Figure 5). The ratios, calculated based on nitric oxide (NOx) production, between HT29 and SW620 cell lines stimulated with *E. coli* 0111:B4 LPS only and LPS in combination with WSP are presented in Table 1.

The results presented in Table 1 indicate whether LPS itself has an impact on NOx levels, which would represent the usual bacterial infection that occurs in patients with colorectal cancer and the role of WSP in modulating NOx release. It is shown whether WSP in combination with LPS would stimulate or reduce NOx production in specific cells after a specific incubation time (24 h or 48 h), compared to the activity of LPS added alone. The table shows how the WSP and LPS administered in combination interact with the induction of NOx in the tested cells. We used the NOx level induced only by LPS alone as the reference control to which we compared the interactions of the polymer and LPS administered together. In the table, the interaction effect of WSP and LPS is described as additive, synergistic, or antagonistic compared to the level of NOx stimulated only by LPS.

**Figure 6:**
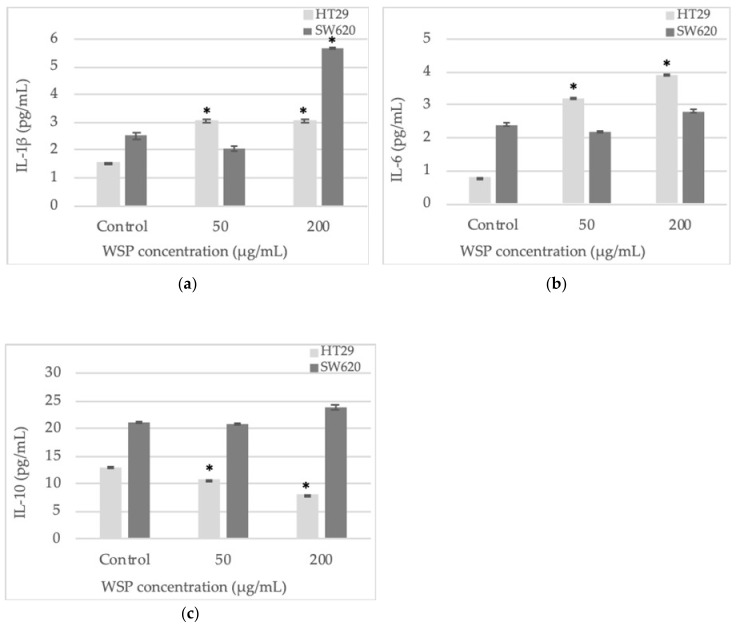
IL-1β (**a**), IL-6 (**b**), and IL-10 (**c**) secretion assessed by ELISA in the culture of HT29 and SW620 cells after 24-h incubation with water-soluble polysaccharide isolated from *A. heterophyllus*. Two concentrations of the compound were used: 50 µg/mL and 200 µg/mL. * *p* ≤ 0.05 - cells treated with the plant polymer compared to the non-treated culture control.

The analysis of the cytokine level was carried out after 24-h incubation of the cells with the WSP at concentrations of 50 or 200 µg/mL. The HT29 cells produced significantly higher amounts of IL-1β after the incubation with both low and high concentrations of the tested polysaccharide, compared to the untreated control. The SW620 cells were stimulated to intensive production of this cytokine only by 200 µg/mL of WSP but it was almost 2-times higher (5.7 pg/mL) than that observed in the HT29 cells (Figure 6a).

The IL-6 level increased in a WSP concentration-dependent manner only in the HT29 cell culture (Figure 6b). On the other hand, the level of IL-10 decreased in a polysaccharide concentration-dependent manner also only in the HT29 cell culture (Figure 6**c**). The SW620 cells were basically not significantly stimulated by the tested polymer to produce IL-6 and IL-10 (Figure 6b,c).

### 2.3. WSP Reducing Activity 

The tested water-soluble polysaccharide showed strong concentration-dependent DPPH and Fe^3+^ reducing activity. Due to the fact that water-soluble polysaccharide from *A. heterophyllus* pulp contains only a small amount of phenols (0.47%) [22], we did not determine the level of this factor in our preparation. In the case of DPPH, the activity of the highest WSP concentration (250 µg/mL) corresponded to 16.2 µg/mL of Trolox, while in the case of the FRAP method it was equivalent to 48.4 µg/mL of ascorbic acid (Table 2 and Table 3).

## 3. Discussion

Polysaccharides, which are high molecular-weight compounds, have been found to possess many biological activities, including cell proliferation modulatory, anti-tumor, antioxidant, anti-inflammatory, or immunostimulatory effects [18]. In the present study, we attempted to find out whether water-soluble polysaccharide isolated from *A. heterophyllus* can influence the viability of human colon carcinoma cells and change pro- (IL-1β, IL-6) and anti-inflammatory (IL-10) cytokines and nitric oxide (NO) produced by them. Moreover, we performed biochemical analyses to determine the free radical scavenging activity of these compounds. 

Polysaccharides are regarded to be polymers with low toxicity exerting few side effects if applied in vivo [32]. In our study, we found that water-soluble polysaccharide from the *A. heterophyllus* exerted very low toxic effects on the viability and metabolic activity of human colon carcinoma cells.

However, the higher concentrations (>225 µg/mL) of the compound limited the metabolism of the cancer cells slightly but statistically significantly. This is in agreement with observations reported by Fan et al. [33], who found that a high concentration of a polysaccharide from *Sargassum fusiforme* (Harv.) Setchel had cytotoxic activity against a liver cancer cell line (HepG2). The finding of our research, however, is the demonstration of the cytotoxic effect at concentrations of the polysaccharide (up to 250 µg/mL), whereas Fan et al. [33] showed this effect using almost 10 times higher concentrations. On the other hand, using a similar range of concentrations as those applied in our study, Hu et al. [34] showed an anticancer effect of a *Chenopodium quinoa* polysaccharide fraction against human normal liver (L02) cells, human normal breast epithelial (MCF10A) cells, human liver cancer (SMMC 7721) cells, and human breast cancer cells (MCF-7). Similar results were also observed after application of a high concentration (up to 4.5 mg/mL) of water-soluble polysaccharides from *Enteromorpha tubulosa* in a study on human breast cancer cell line MCF-7 in vitro [34]. It was also revealed that polysaccharides might enhance the antitumor effect when combined with chemotherapeutic drugs [35]. This may therefore suggest that the anticancer cytotoxic effect strongly depends not only on the polymer concentration used but also on the origin and structure of the compound. Moreover, the activity of the polysaccharide compound may also exert different cytotoxic effects depending on the type of cells. For example, a sulfated *Artemisia sphaerocephala* polysaccharide inhibited the growth of human liver and cervical carcinomas but had no effect against mouse adipocyte normal fibroblasts (L929) [36].

Other important bioactivities that can be regarded as characteristic for many plant polysaccharides are their anti-oxidative and immunomodulatory effects. In terms of the anti-oxidative abilities of the tested polysaccharides, we analyzed the ability to reduce free radicals and changes in nitric oxide (NOx) release by the cultured cells in vitro. In our study, the NOx release was dependent on the concentration and time of incubation with the compound. Generally, the tested polymer induced NOx release by the cancer cells and additionally exhibited significant activities, i.e., a reduction of reactive oxygen species (ROS), in our tests of DPPH radical. It may be suggested that the polymer can activate transcription factors like NF-κB or AP-1 and thus induce NOS activity and, consequently, NOx production. Polysaccharides isolated from *Plantago palmata* leaves or a polysaccharide isolated from *Glycyrrhiza uralensis* and ascophyllan, i.e., a fucose-containing sulfated polysaccharide from the brown alga *Ascophyllum nodosum*, stimulated NOx release by macrophages [20,37,38]. Moreover, a sulfated polysaccharide fraction from the alga *Hypnea musciformis* stimulated NOx release by neutrophils as well [19]. In contrast, polysaccharides isolated from *Phellinus baumii* significantly reduced NOx release by Abelson murine leukemia virus-transformed macrophages [21]. However, this observation was not confirmed in the study conducted by Diao et al. [39], who applied *Bletilla striata* polysaccharides to the same cells. These results indicate that, depending on the type and origin of polysaccharides, as well as the time and concentrations used, different results can often be obtained. This also indicates a smooth border between the beneficial, neutral, or adverse activities of plant polysaccharides on human and animal cells. Generally, plant polysaccharides are indicated to have anti-oxidative activities. A water-soluble polysaccharide from *A. heterophyllus* Lam. (jackfruit) pulp was found to scavenge DPPH• and •OH radicals potently [22], likewise a polysaccharide derived from *Cordyceps sinensis* [23]. Other studies analyzing the use of polysaccharides from various plants also indicate the high antioxidant potential of these compounds [18,34]. In our study, we confirmed such observations. These properties can be taken into account and potentially applied in medicine or food industry.

The immunomodulating activities of polysaccharides isolated from different plants have also been shown. In our study, we found increased levels of IL-1β and IL-6 depending on the cell culture and polysaccharide concentration. On the other hand, no changes or reduction of the level of anti-inflammatory IL-10 were detected. These results are consistent with observations reported by Biringanine et al. [37], who found that polysaccharides from *Plantago palmata* leaves induce TNF-α release but reduce IL-10 production by IFN-γ-activated C3H/Hej macrophages. In addition, WSP limited the amount of IL-10 detected in HT29 cells isolated from an early stage of tumor development and increased the amount of this cytokine released by SW620 cells obtained from a colon tumor. The obtained results can be explained in such a way that the tested polymer would work favorably, in a desirable way, but only in the early stages of the disease. It would limit immunosuppression and thus slow down tumor growth by facilitating its recognition by e.g., infiltrating T effector lymphocytes. In the late stages, WSP action would be opaque because, by enhancing the state of immunosuppression, it would additionally protect proliferating cancer cells from attack by the host’s immune system. Moreover, crude polysaccharides of *Citrus aurantium* L. var. amara Engl stimulated TNF-α and IL-6 release by mouse macrophages [18] and TNF-α by peritoneal macrophages after stimulation with *Dendrobium huoshanense* polysaccharides [24]. These results can potentially be explained by the effect of elevated NOx levels on mRNA for some cytokines, hence the increase in IL-1β or IL-6 (the TNF-α/IL-1β pathway, but not the IL-10 pathway) in various cell types.

## 4. Materials and Methods 

### 4.1. Isolation of the Water-Soluble Polysaccharide from A. heterophyllus Fruits

The water-soluble polysaccharide was isolated from mature *A. heterophyllus* fruits using a modified procedure proposed by Das and Rao [40]. Briefly, the outer skin of the fruits was removed and the sliced mesocarp was macerated for 2 min in a blender in the presence of 96% ethanol and allowed to stand overnight. The suspension was filtered through a 0.45 µm membrane of a Vacuum Driven Disposable Filtration System (Durapore, Millipore, Billerica, MA, USA) and air-dried at 45 °C to yield a fibrous material. The material obtained (50 g) was stirred with Milli-Q water (2 L) at boiling water-bath temperature for 5 h and the resulting slurry was allowed to stand overnight. Next, it was filtered through a 0.45 µm membrane and the water extract was clarified by centrifugation (17,001× *g* for 30 min). The clear solution was added to acidified (pH 4) 96% ethanol (4 L) when a white gelatinous precipitate formed. The precipitate was collected by centrifugation (17,001× *g* for 30 min), washed with 96% ethanol, and lyophilized to obtain a white powder.

### 4.2. Carbohydrate Analysis

For monosaccharide analysis, the polysaccharide was hydrolyzed with 2 M CF_3_CO_2_H (100 °C, 4 h). The absolute configuration of monosaccharides was established by an analysis of acetylated *R*-(−)2-butylglycosides according to Gerwig and co-workers [41]. The sugars were converted into alditol acetates [42]. The water-soluble material was methylated according to the method developed by Hakomori [43], and the methylated polysaccharide was purified on a Sep-Pak C18 cartridge [44]. The resulting material was carboxyl reduced with NaBD_4_ and finally hydrolyzed in 2 M CF_3_CO_2_H (100 °C, 4 h) and again reduced with NaBD_4_ [44]. Partially methylated alditols were converted to acetate derivatives. The alditol acetates and the partially methylated alditol acetates were analyzed using combined gas chromatography–mass spectrometry (GC–MS). The GC–MS analyses were carried out on a Hewlett-Packard gas chromatograph (model HP5890A) equipped with a capillary column (HP-5MS, 30 m × 0.25 mm) and connected to a mass selective detector (MSD model HP5971). Helium was the carrier gas, and the temperature program was 150 °C initially for 5 min. Next, it was raised to 310 °C at a ramp rate of 3 °C/min, and the final time was 20 min. The molecular weight of β-galactan was determined by high performance gel permeation chromatography on a Shimadzu HPLC system equipped with a PolySep™-SEC GFC-P 5000 gel-filtration chromatographic column (300 × 7.8 mm) and detected by a RID-10A refractive index detector (Shimadzu, Kyoto, Japan) set at 30 °C. Milli-Q water was used as the mobile phase at a flow rate of 0.8 mL/min. The column was calibrated with a set of dextran standards (Sigma, St. Louis, MO, USA). 

An infrared absorption spectrum (FT-IR) between 400 and 4000 cm^−1^ was recorded using a Perkin Elmer FT-IR spectrophotometer (Model 1725X). A specimen was prepared by the KBr-disk method. The degree of esterification (DE) was calculated from FTIR spectra using the method described by Manrique and Lajlo [45]. The following equation was used: DE = [A_1737_/(A_1737_ + A_1606_] × 100%.

Specific rotation [α]${}_{D}^{25}$(*c* 1.0, H_2_O) was measured at 589 nm in a Perkin Elmer Automatic Polarimeter (Model 341 LC). The viscosity of the polysaccharide (*c* 1.0, H_2_O) was measured with a Brookfield (Model DV 3) viscometer at 20 °C. 

### 4.3. Cell Culture

Human colon adenocarcinoma cell lines HT29 (ATCC No. HTB-38) derived from grade I tumor and SW620 (ATCC No. CCL-227) from a metastatic site in a lymph node (Duke’s C) were cultured in RPMI 1640 medium supplemented with 10% fetal calf serum (FCS) (Gibco^TM^, Paisley, UK) and antibiotics (100 U/mL penicillin and 100 µg/mL streptomycin) (Sigma, St. Louis, MO, USA) at 37 °C in a humidified atmosphere with 5% CO_2_.

### 4.4. Neutral Red (NR) Uptake Assay

The NR cytotoxicity assay is based on the uptake and lysosomal accumulation of the supravital dye Neutral Red. Cells with damaged membranes do not take up the dye. Cells (1 × 10^5^ cells/mL in 100 µL of medium RPMI 1640) after 24-h adhesion in 96-well multiplates were subsequently grown in 100 µL of culture medium and at various concentrations of *A. heterophyllus* WSP (25–250 µg/mL) for 24 and 48 h. After that time, the plate was placed for 3 h at 37 °C in a humidified 5% CO_2_/95% air incubator. After incubation, the dye-containing medium was removed, the cells were fixed with 1% CaCl_2_ in 4% paraformaldehyde, and the incorporated dye was solubilized using 1% acetic acetate in a 50% ethanol solution (100 µL). The plates were gently shaken for 20 min at room temperature and the dye absorbance was measured spectrophotometrically at 540 nm using an EL800 Universal Microplate Reader (BioTek Instruments, Winooski, VT, USA).

### 4.5. MTT Assay

The MTT test is based on the conversion of a yellow tetrazolium salt by viable cells to purple crystals of formazan. Mitochondrial succinyl dehydrogenase catalyzes this reaction. Cell sensitivity to *A. heterophyllus* WSP was determined using a standard spectrophotometric 3-(4.5-dimethylthiazole-2-yl)-2.5-diphenyltetrazolium bromide (MTT) assay. After 24-h adhesion in 96-well multiplates, the cells (1 × 10^5^ cells/mL in 100 µL of medium RPMI 1640) were subsequently grown in 100 µL of culture medium and various concentrations of polysaccharides (25–250 µg/mL) for 24 and 48 h. After that time, an MTT solution (5 mg/mL, 25 µL/well) (Sigma, St. Louis, MO, USA) was added to the medium for 3-h incubation. The yellow tetrazolium salt was metabolized by viable cells into purple formazan crystals. The crystals were solubilized overnight in 10% sodium dodecyl sulfate (SDS) in 0.01 M HCl. The product was quantified spectrophotometrically by reading the absorbance at 570 nm using an EL800 Universal Microplate Reader (BioTek Instruments, Winooski, VT, USA).

### 4.6. Nitric Oxide (NOx) Measurement

Nitrate, i.e., a stable end product of nitric oxide, was determined in culture supernatants by a Griess reaction. Briefly, cells (1 × 10^5^ cells/mL in 1 mL of medium RPMI 1640) after 24-h adhesion in 24-well plates were induced with four concentrations of *A. heterophyllus* WSP (50, 100, 150, and 200 µg/mL) for 24 and 48 h. After that time, 100 µL of culture supernatant was placed in 96-well plates in triplicate and incubated with 100 µL of Griess reagent (1% sulfanilamide/0.1% *N*-(1-naphthyl) ethylenediamine dihydrochloride) (Sigma, St. Louis, MO, USA) in 3% H_3_PO_4_ (POCH Gliwice, Poland) at room temperature for 10 min. The optical density was measured at 550 nm using an EL800 Universal Microplate Reader (BioTek Instruments, Winooski, VT, USA). A standard curve was achieved using 0.5–25 µM sodium nitrite (NaNO_2_) for calibration. For the analysis of the NOx production, the cells were cultured in standard conditions and incubated with *A. heterophyllus* WSP. In parallel tests, the cells were pre-activated for 2 h with 10 µg/mL LPS from *Escherichia coli*, serotype 0111:B4, and then incubated with the polysaccharide for 24 or 48 h.

### 4.7. DPPH• Free Radical Scavenging Test

The free radical scavenging activity of *A. heterophyllus* WSP was measured by the 1.1-diphenyl-2-picrylhydrazyl (DPPH•) assay, where the ability of antioxidants to reduce the stable dark violet radical DPPH• (Sigma, St. Louis, MO, USA) to the yellow diphenyl-picrylhydrazine is analyzed. Briefly, 100 µL of a DPPH• solution (0.2 mg/mL in ethanol) was added to 100 µL of the WSP concentrations (25–250 µg/mL) and standards. Trolox (Sigma, St. Louis, MO, USA) at increasing concentrations (1–50 µg/mL) was used as a reference for the free radical scavenging activity. After 20-min incubation at room temperature, the absorbance was measured at 515 nm using an EL800 Universal Microplate Reader (BioTek Instruments, Winooski, VT, USA). Lower absorbance indicates greater free radical scavenging activity of the WSP. The activity of WSP was determined by comparing its absorbance with that of a blank solution (reagents without WSP) and a standard. The capability to scavenge DPPH• radical was calculated with the following formula:

DPPH• scavenging effect (%) = [(X*control* − X*WSP* /X*control*) × 100]

where X*control* is the absorbance of the control and X*WSP* is the absorbance in the presence of WSP.

### 4.8. Ferric-Reducing Antioxidant Power Assay (FRAP)

Each polysaccharide concentration was dissolved in Milli-Q water and mixed with an equal volume of 0.2 M sodium phosphate buffer (pH 6.6) and 1% potassium ferricyanide. The mixture was incubated for 30 min at 37 °C. Thereafter, 10% trichloroacetic acid (*w*/*v*) was added and the mixture was centrifuged at 1000× *g* for 5 min. One ml of the upper layer was mixed with an equal volume of Milli-Q water and 0.1% ferric chloride. The absorbance was read at 700 nm using an EL800 Universal Microplate Reader (BioTek Instruments, Winooski, VT, USA). Ascorbic acid (0–150 µg/mL) was used as a positive control.

### 4.9. ELISA Assay

The levels of human IL-1β, IL-6, and IL-10 were tested immunoenzymatically (ELISA) using commercially available kits (BD OptEIATM, San Jose, CA, USA) according to the manufacturer’s instructions. The optical density at 450 nm with a correction wavelength of 570 nm was determined for each ELISA sample using an EL800 Universal Microplate Reader (BioTek Instruments, Winooski, VT, USA). The cytokine concentrations were calculated on the basis of a standard curve. The detection limits were 2 pg/mL (IL-1β), 2.2 pg/mL (IL-6), and 2 pg/mL (IL-10).

### 4.10. May-Grünwald-Giemsa (MGG) Staining

The MGG staining performed in this study revealed morphological changes in the cells induced by the *A. integrifolia* WSP at the concentration of 250 µg/mL. 

Cells at a density of 1 × 10^5^ cells/mL were cultured in Petri dishes (35 mm). After 24-h treatment with *A. heterophyllus* WSP, the medium was discarded and the MGG staining protocol was initiated. The cells were stained and fixed with the May-Grünwald dye for 2 min and for another 2 min in a dye diluted with an equal volume of water. Thereafter, the dye was removed and Giemsa stain, previously diluted (1 *vol*. Giemsa: 19 *vol*. water), was added for 20 min. The dishes were rinsed three times with distilled water and dried. The observation was performed under a light microscope (Olympus BX51).

### 4.11. Statistical Analysis

The results are presented as means ± SD of three independent experiments (n = 3). The data were analyzed using one-way analysis of variance ANOVA followed by Dunnett’s multiple comparison post-hoc test. Differences of *p* ≤ 0.05 were considered significant. 

## 5. Conclusions

The results presented and discussed in this study indicate that, generally, plant-derived polysaccharides and, more specifically, water-soluble polysaccharides isolated from *A. heterophyllus* exhibit significant biological activity towards many types of both normal and cancerous cells. In our study, the tested *A. heterophyllus* compounds had no strong toxicity to human colon tumor cells but exhibited immunomodulatory activity as well as significant anti-oxidative effects. Therefore, an important finding of our research was the demonstration that relatively low concentrations of the tested polymer (< 250 µg/mL) expressed quite high biological activity. This may indicate that the compound has beneficial properties in medicine. It may also be used as a supplementary compound in the food industry, which is closely connected with its pro-health features.

## Figures and Tables

**Table 1:** Ratios, calculated based on nitric oxide (NOx) production, between HT29 and SW620 cell lines stimulated with *E. coli* 0111:B4 LPS only and LPS in combination with WSP.

Cell Culture and Time of Incubation (h) LPS +	WSP (µg/mL)			
	50	100	150	200
HT29 (24 h)	Antagonism 2.57	Additivity 1.0	Additivity 0.71	Synergism 0.39
SW620 (24 h)	Antagonism 13.25	Synergism 0.68	Antagonism 3.75	Antagonism 2.38
HT29 (48 h)	Additivity 0.84	Additivity 0.91	Additivity 0.87	Additivity 0.72
SW620 (48 h)	Additivity 0.8	Antagonism 1.8	Antagonism 2.0	Synergism 0.33

Quotients lower than 0.7 indicate synergism of reciprocal interaction of the tested compounds on NOx production by human colon cancer cells; quotients higher than 1.3 indicate antagonism of interactions; quotients in the range of 0.7–1.3 indicate additive relations. The ratio represents the mutual interactions between *E. coli* 0111:B4 LPS and the polysaccharide in stimulation of the production of nitric oxide (NOx) by the tested cells at a specified incubation time.

**Table 2:** DPPH free radical scavenging activity. The percentage of reduced DPPH radicals by WSP isolated from *A. heterophyllus* is compared to the control (0% reduction).

WSP (µg/mL)	% of Reduction Compared to the Control	Reduction Value Corresponding to Trolox Concentrations (µg/mL)
25	18.3 * ± 0.2	10.6
50	21.4 * ± 0.2	12.4
75	24.0 * ± 0.3	14.0
100	24.4 * ± 0.2	14.2
125	25.7 * ± 0.3	15.0
150	26.2 * ± 0.4	15.3
175	26.4 * ± 0.4	15.4
200	26.8 * ± 0.2	15.6
225	26.9 * ± 0.2	15.7
250	27.7 * ± 0.4	16.2

* *p* ≤ 0.05 - cells treated with the plant polymer compared to the non-treated culture control.

**Table 3:** Ferric-reducing antioxidant power assay. The percentage of reduced ferric ions by WSP isolated from *A. heterophyllus* is compared to the control (0% reduction).

WSP (µg/mL)	% of Reduction Compared to the Control	Reduction Value Corresponding to Ascorbic Acid Concentrations (µg/mL)
25	44.8 * ± 0.1	9.9
50	44.8 * ± 0.1	9.9
75	46.8 * ± 0.1	11.0
100	48.5 * ± 0.1	12.1
125	48.7 * ± 0.1	12.3
150	57.6 * ± 0.1	18.6
175	59.5 * ± 0.1	20.3
200	68.4 * ± 0.1	31.1
225	73.6 * ± 0.1	41.0
250	76.5 * ± 0.1	48.4

* *p* ≤ 0.05 - cells treated with the plant polymer compared to the non-treated culture control.
